# Clinicopathological Profile and Diagnostic Concordance of Skin Adnexal Tumours at a Tertiary Care Centre

**DOI:** 10.7759/cureus.106069

**Published:** 2026-03-29

**Authors:** Gayathri Priyadharshini, Manjiri Phansalkar, Kiran Mahesh, Anita Ramdas

**Affiliations:** 1 Pathology, Pondicherry Institute of Medical Sciences, Puducherry, IND; 2 Medical Education, Northern Territory Medical Program, Flinders University, Darwin, AUS

**Keywords:** clinicopathological profile, diagnostic concordance, eccrine, follicular, histopathology, sebaceous, skin adnexal tumours

## Abstract

Background: Skin adnexal tumours are a morphologically heterogeneous group of neoplasms arising from cutaneous appendages. Their clinical similarity to common benign lesions renders preoperative diagnosis challenging, and systematic concordance data from Indian tertiary institutions remain limited.

Objectives: The objectives of this study are to characterise the histopathological spectrum and clinicopathological associations of skin adnexal tumours and to quantify clinical-histopathological concordance.

Methods: A cross-sectional, descriptive-analytical study was conducted at a tertiary care teaching hospital over six years using consecutive sampling. Data were analysed using descriptive statistics, Fisher's exact test, Chi-square test, Mann-Whitney U test, and Cohen's Kappa coefficient.

Results: Forty-seven cases were studied (M:F = 1.47:1; mean age 42.32 ± 15.37 years). Eccrine differentiation predominated (66.0%), followed by follicular (23.4%) and sebaceous (10.6%) lineages. The consolidated Hidradenoma group was the most frequent individual diagnosis (17.0%), followed by pilomatricoma (10.6%). Benign tumours constituted 83.0%, whereas malignant tumours constituted 17%; the head and neck was the predominant site (44.7%). No clinicopathological variable was significantly associated with biological behaviour. The overall concordance rate was 21.3% (Cohen's κ = −0.575; 95% CI: −0.792 to −0.357; poor agreement). Differentiation type (χ² = 11.655, p = 0.003) and anatomical site (χ² = 7.881, p = 0.049) were the only significant concordance predictors; sebaceous tumours (80.0%; OR = 28.0) and head and neck location (38.1%) showed the highest rates, while extremity tumours were uniformly discordant (0%).

Conclusion: A 78.7% discordance rate confirms the indispensability of histopathological examination for all excised adnexal lesions. Sebaceous differentiation and head-and-neck location predict better clinical recognition; extremity lesions require heightened diagnostic vigilance.

## Introduction

Skin adnexal tumours constitute a morphologically diverse group of neoplasms arising from the pilosebaceous unit and the eccrine and apocrine sweat glands of the skin [1-3]. They encompass a wide biological spectrum, from entirely benign hamartomas and adenomas to frankly malignant carcinomas, with several intermediate entities of uncertain biological behaviour [1]. Despite their relative infrequency in routine surgical pathology practice, accurate histopathological classification carries critical implications for surgical planning, margin adequacy, prognostication, and long-term patient surveillance [2,3].

The global literature documents considerable variation in the frequency, histological distribution, and demographic associations of adnexal tumours across geographic regions and ethnic populations [4-8]. Reports from India and South Asia have documented certain consistent patterns, including eccrine predominance, male preponderance, and a comparatively lower frequency of malignant sebaceous tumours compared to Western series, yet published institutional data remain sparse, often limited by small sample sizes, incomplete histological characterisation, or absence of systematic concordance analysis [4-9]. Prior Indian studies have provided valuable descriptive baseline data on histological subtype distribution and demographic associations but have not systematically addressed the clinical-histopathological concordance dimension [7-10].

A critical yet underexplored dimension of adnexal tumour management is clinical-histopathological concordance, the degree to which preoperative clinical impressions agree with the definitive histopathological diagnosis. These tumours frequently masquerade as sebaceous cysts, lipomas, dermoid cysts, lymph nodes, or unrelated malignant lesions, leading to misclassification and potentially inappropriate clinical decisions [2,3]. The consequences of diagnostic discordance include under-treatment of malignant lesions initially believed to be benign, failure to ensure adequate surgical margins, unnecessary patient anxiety, and misdirected investigations. While isolated reports have highlighted diagnostic pitfalls for individual tumour types [11-13], a systematic institutional analysis quantifying overall concordance across the full adnexal tumour spectrum, with Kappa-based statistical rigour [12-15], and identification of concordance predictors, has not been previously published from an Indian tertiary centre.

The present study addresses this research gap through a comprehensive six-year clinicopathological analysis, and the novelty lies in its integrated approach: simultaneously characterising the histopathological spectrum, evaluating clinicopathological associations, and systematically quantifying clinical-histopathological concordance using Cohen’s Kappa [12], with identification of independent concordance predictors. This integrated framework provides a clinically actionable, statistically robust dataset that contributes institution-specific and region-specific evidence to the pathological literature on skin adnexal tumours.

## Materials and methods

Research setting

This study was conducted in the Department of Pathology at a tertiary care teaching hospital that serves as a major referral centre with a wide geographic catchment area. The study was conducted in accordance with the Declaration of Helsinki [14] and received Institutional Ethics Committee approval prior to data collection. Patient confidentiality was maintained through complete de-identification of all data.

Research design

A cross-sectional, descriptive-analytical design was employed, enabling simultaneous characterisation of the clinicopathological profile and quantification of diagnostic concordance through an inferential statistical test.

Study duration and sampling

All histopathologically confirmed skin adnexal tumour cases over a consecutive six-year period were eligible. Consecutive sampling constituted a total enumeration study, minimising selection bias. No a priori sample size calculation was performed, as the study intended to capture all eligible cases within the defined period.

Eligibility criteria

Inclusion required (i) a final histopathological diagnosis of a primary skin adnexal tumour and (ii) adequate clinical data, including age, sex, anatomical site, presenting complaint, and preoperative clinical diagnosis. Cases were excluded for inadequate histopathological material, secondary or metastatic adnexal differentiation, or missing clinical records precluding concordance assessment.

Data collection

A structured proforma captured (a) demographics: age and sex; (b) clinical data: anatomical site (head and neck, trunk, upper extremity, lower extremity), number of lesions, presenting complaint, and pain; (c) preoperative clinical diagnosis and (d) histopathological data: final diagnosis, differentiation type (eccrine/follicular/sebaceous), biological behaviour (benign/malignant) and histological location.

Histopathological processing and classification

Specimens were fixed in 10% neutral buffered formalin, paraffin-embedded, sectioned at 3-5 μm, and stained with haematoxylin and eosin. Special stains were applied selectively. Classification followed the WHO Classification of Skin Tumours (4th edition, 2018) [1], with multidisciplinary departmental consensus for uncertain cases.

Assessment of diagnostic concordance

Concordance was defined as agreement between the preoperative clinical and final histopathological diagnoses. Cases with absent preoperative diagnosis were classified as discordant. Clinically reasonable approximations were accepted as Concordant when the clinical label reflected the same or a closely related tumour category. Entirely unrelated diagnoses were classified as Discordant.

Statistical analysis

Data was analysed using IBM SPSS Statistics for Windows, Version 25 (Released 2017; IBM Corp., Armonk, New York, United States). Categorical variables were expressed as frequency and percentage with 95% CI by the Wilson score method: continuous variables as mean ± SD with 95% CI by the t-distribution formula. Normality was assessed by the Shapiro-Wilk test. Fisher's exact test was applied for 2×2 and sparse tables; Pearson's Chi-square test was applied for tables with expected cell frequencies≥5. Mann-Whitney U and Kruskal-Wallis tests were used for nonparametric comparisons. Odds ratios with 95% CI were calculated for binary associations, and concordance was quantified by Cohen's Kappa (κ). A two-tailed p < 0.05 was considered statistically significant [12].

## Results

Sociodemographic and clinical profile

**Figure 1:**
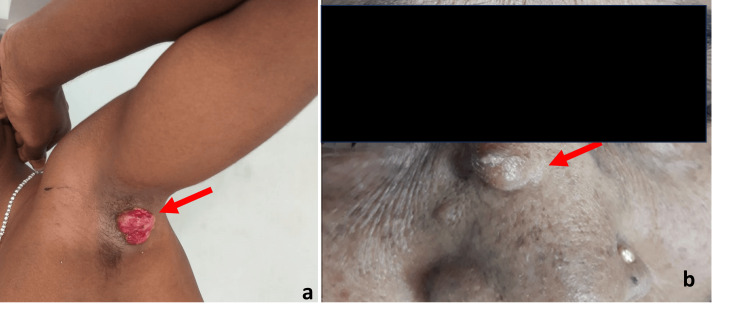
Clinical photograph showing (a) a solitary, well-circumscribed nodular swelling with ulceration on the skin surface and (b) a well-defined, firm, nodular swelling over the skin surface with smooth overlying skin on the face.

A total of 47 histopathologically confirmed skin adnexal tumours were analysed. A modest male predominance was observed (M: F ratio 1.47:1; 59.6% vs. 40.4%), with a mean age of 42.32 ± 15.37 years (95% CI: 37.81-46.83 years) and an age range of 2-75 years (Table 1). The 41-60-year group was the most represented (44.7%; 95% CI: 30.5-58.9%). Clinically, swelling (Figure 1) was the predominant presenting complaint (59.6%), and the majority of lesions were painless (76.6%) and solitary (74.5%).

**Table 1:** Sociodemographic and clinical profile of patients with skin adnexal tumours (N = 47).

Characteristic	n (%) or Value	95% CI
Sex		
Male	28 (59.6%)	45.5 – 73.7%
Female	19 (40.4%)	26.3 – 54.5%
Male:Female ratio	1.47:1	—
Age (years)		
Mean ± SD	42.32 ± 15.37	37.81 – 46.83 years
Median (IQR)	44.0 (30.0 – 54.0)	—
Range	2 – 75 years	—
Age Group (years)		
1 – 20	3 (6.4%)	0.0 – 13.4%
21 – 40	16 (34.0%)	20.5 – 47.5%
41 – 60	21 (44.7%)	30.5 – 58.9%
61 – 80	7 (14.9%)	4.8 – 25.0%
Presenting Complaint		
Swelling	28 (59.6%)	45.5 – 73.7%
Papules/Nodules	7 (14.9%)	4.8 – 25.0%
Swelling with ulceration	6 (12.8%)	3.1 – 22.5%
Other (warty/exophytic/ulceroproliferative)	6 (12.8%)	3.1 – 22.5%
Pain		
Present	11 (23.4%)	11.3 – 35.5%
Absent	36 (76.6%)	64.5 – 88.7%
Number of Lesions		
Single	35 (74.5%)	61.5 – 87.5%
Multiple	12 (25.5%)	13.2 – 37.8%

**Histopathological spectrum and lineage distribution

**Figure 2:**
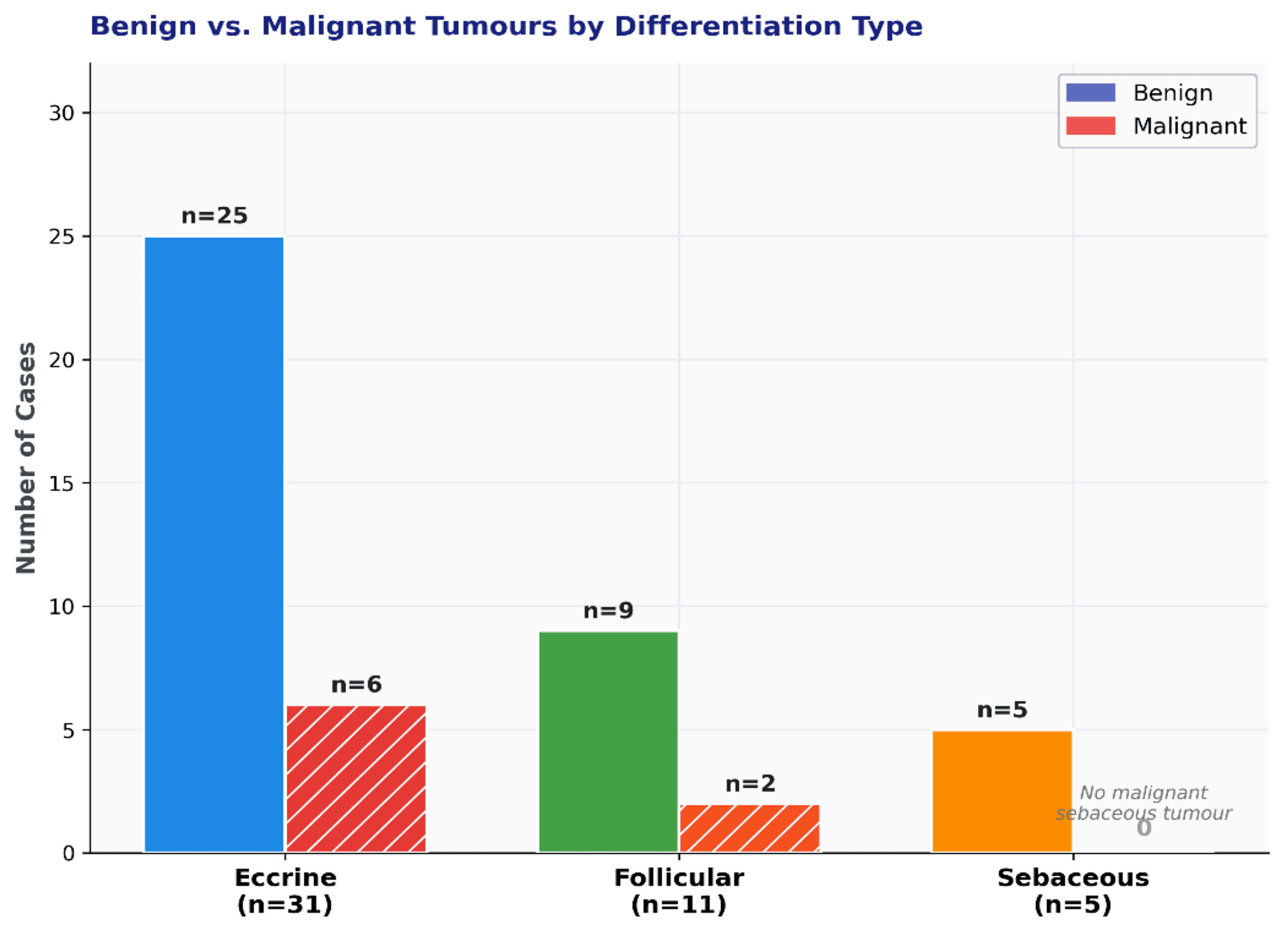
Bar diagram showing the distribution of benign and malignant skin adnexal tumours according to the line of differentiation (eccrine, apocrine, sebaceous, and follicular)

**Figure 3:**
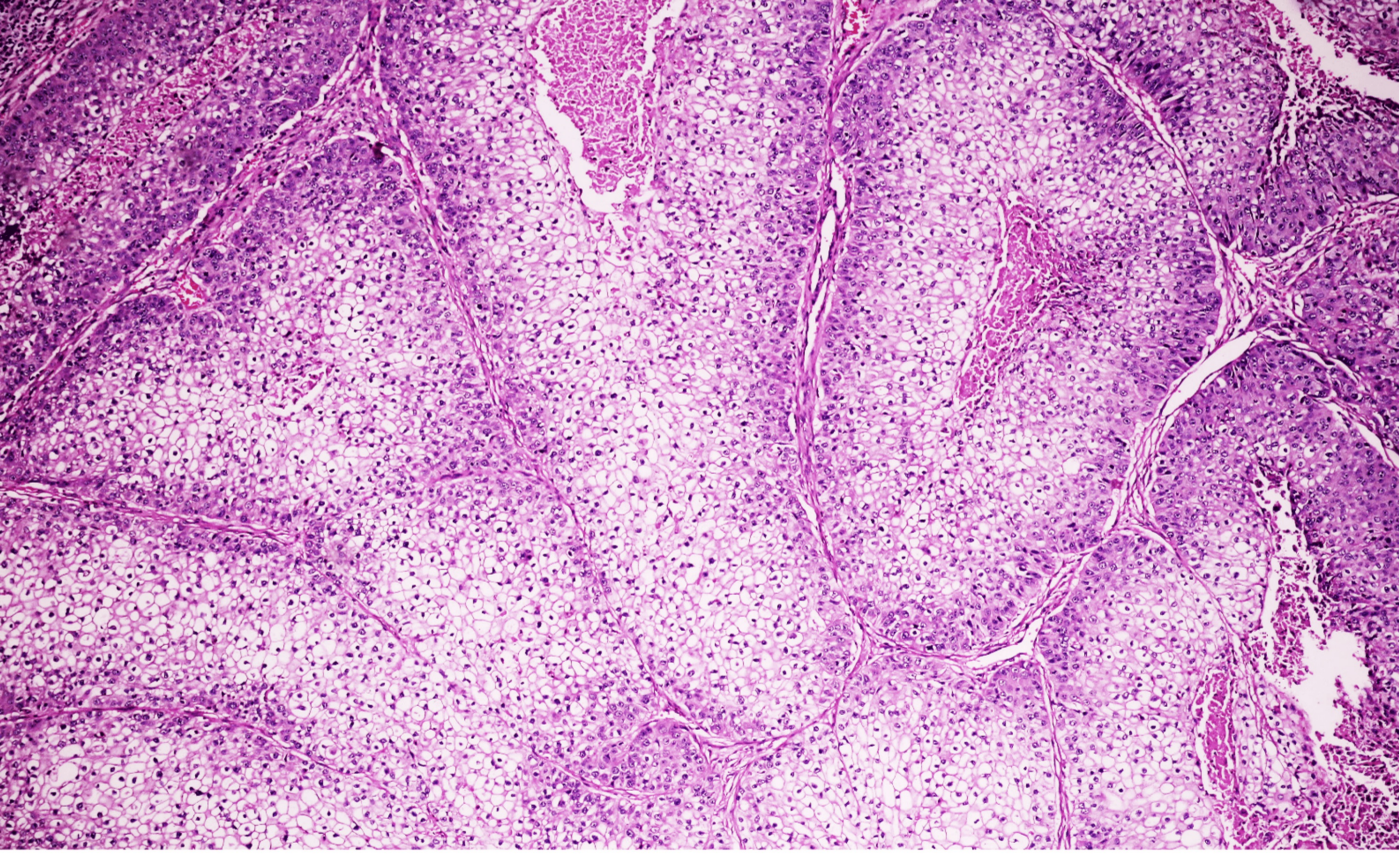
Higher magnification showing two distinct cell populations, polyhedral eosinophilic cells and clear cells with glycogen-rich cytoplasm in a case of Hidradenoma (H&E, ×400).

**Figure 4:**
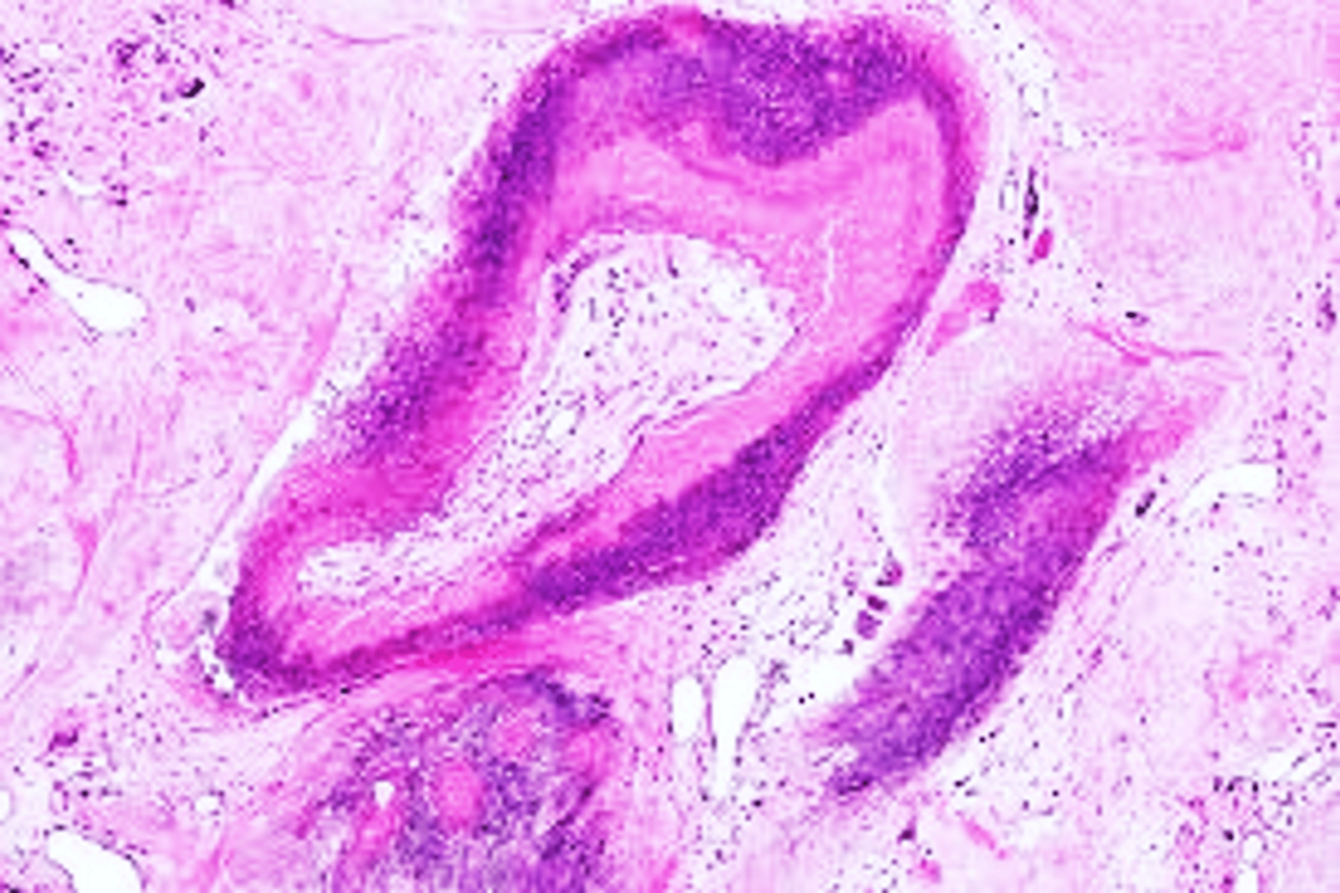
Low-power view showing Pilomatricoma: Islands of basophilic cells, shadow (ghost cells) that consist of eosinophilic keratinised cytoplasm without nuclei (H&E, x100).

**Figure 5:**
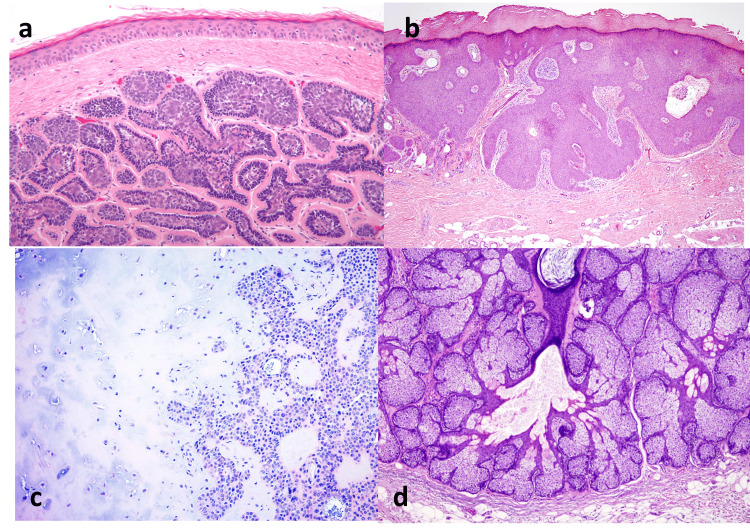
Photomicrograph showing (a) dermal tumour with islands arranged in a characteristic “jigsaw puzzle” pattern, consistent with cylindroma (H&E, x100), (b) dermal tumour composed of broad anastomosing bands of epithelial cells extending from the epidermis, characteristic of eccrine poroma (H&E, x100), (c) tumour demonstrating branching tubules, cords, and nests of epithelial cells embedded within abundant chondromyxoid stroma, characteristic of chondroid syringoma (H&E, ×100), and (d) sebaceous adenoma showing lobulated sebaceous proliferation with peripheral basaloid cells and central mature sebocytes containing vacuolated cytoplasm (H&E, ×100).

**Figure 6:**
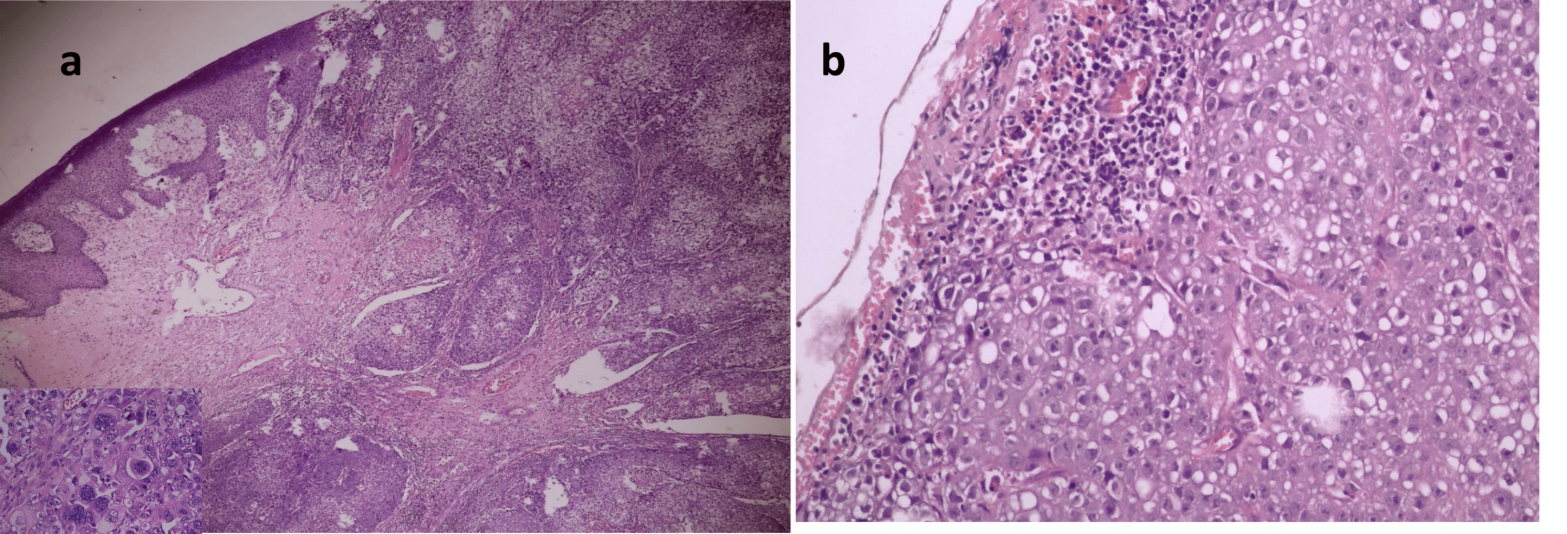
(a) Histopathological section of a cutaneous tumour showing infiltrative nests and cords of atypical epithelial cells with ductal differentiation and two distinct populations with nuclear pleomorphism (inlet image) in the dermis. (b) High-power view of lymph node metastasis highlighting pleomorphic tumour cells arranged in nest-like structures (H&E stain, ×400).

**Figure 7:**
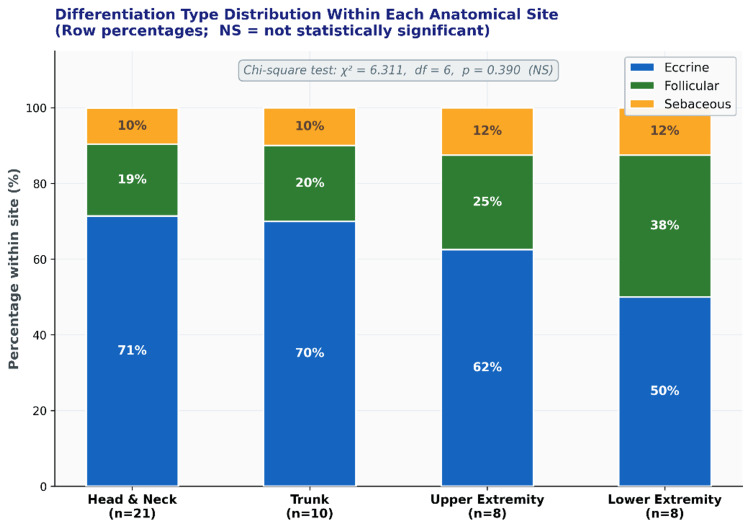
Bar diagram showing the number of skin adnexal tumor cases across different anatomical sites.

Twenty-two distinct diagnoses were identified. Eccrine differentiation predominated (66.0%; 95% CI: 51.8-80.2%), followed by follicular (23.4%; 95% CI: 11.3-35.5%) and sebaceous lineages (10.6%; 95% CI: 1.9-19.3%) (Table 2; Figure 2). The Hidradenoma (Figure 3) group was the most frequent individual diagnosis (n=8; 17.0%; 95% CI: 8.9-30.1%), followed by Pilomatricoma (Figure 4) (n=5; 10.6%) and Eccrine Spiradenoma (n=4; 8.5%). Benign tumours (Figure 5) predominated (83.0%; n=39); all eight malignancies (Figure 6) were of eccrine (n=6) or follicular (n=2) origin, with no malignant sebaceous tumour identified. Eccrine tumours constituted the dominant lineage at every anatomical site (50-71%), though follicular tumours formed a proportionally larger representation at the extremities (25-38%) than at the head and neck or trunk (19-20%); this distribution did not reach statistical significance (χ² = 6.311, df = 6, p = 0.390) (Figure 7).

**Table 2:** Frequency and distribution of histopathological diagnoses of skin adnexal tumours (N = 47).

Histopathological Diagnosis	Behaviour	n	%	95% CI (%)	Concordance n (%)
Eccrine differentiation — n = 31 (66.0%; 95% CI: 51.8–80.2%)					
Hidradenoma (Nodular/clear cell/benign nodular variants)	Benign	8	17	8.9 – 30.1	0 (0.0%)
Eccrine spiradenoma	Benign	4	8.5	0.6 – 16.4	0 (0.0%)
Syringoma	Benign	3	6.4	0.0 – 13.4	2 (66.7%)
Chondroid syringoma	Benign	3	6.4	0.0 – 13.4	0 (0.0%)
Eccrine poroma	Benign	2	4.3	0.0 – 10.1	0 (0.0%)
Syringocystadenoma papilliferum	Benign	2	4.3	0.0 – 10.1	0 (0.0%)
Eccrine poroma (pigmented variant)	Benign	1	2.1	0.0 – 6.2	0 (0.0%)
Naevus sebaceous with syringocystadenoma	Benign	1	2.1	0.0 – 6.2	1 (100.0%)
Cylindroma	Benign	1	2.1	0.0 – 6.2	0 (0.0%)
Malignant eccrine poroma	Malignant	2	4.3	0.0 – 10.1	1 (50.0%)
Malignant nodular Hidradenoma	Malignant	1	2.1	0.0 – 6.2	0 (0.0%)
Clear cell Hidradenocarcinoma	Malignant	1	2.1	0.0 – 6.2	0 (0.0%)
Mucinous carcinoma	Malignant	1	2.1	0.0 – 6.2	0 (0.0%)
Adenoid cystic carcinoma	Malignant	1	2.1	0.0 – 6.2	0 (0.0%)
Follicular differentiation — n = 11 (23.4%; 95% CI: 11.3–35.5%)					
Pilomatricoma	Benign	5	10.6	1.9 – 19.3	0 (0.0%)
Trichoepithelioma	Benign	3	6.4	0.0 – 13.4	1 (33.3%)
Trichoblastoma	Benign	1	2.1	0.0 – 6.2	0 (0.0%)
Trichilemmal carcinoma	Malignant	2	4.3	0.0 – 10.1	1 (50.0%)
Sebaceous differentiation — n = 5 (10.6%; 95% CI: 1.9–19.3%)					
Nevus sebaceous	Benign	3	6.4	0.0 – 13.4	3 (100.0%)
Sebaceous adenoma	Benign	2	4.3	0.0 – 10.1	1 (50.0%)
Total	—	47	100	—	10 (21.3%)

Association of clinicopathological variables with diagnostic concordance

Differentiation type was a significant determinant of concordance (χ² = 11.655, df = 2, p = 0.003). Sebaceous tumours achieved the highest concordance rate (80.0%; 4/5), markedly exceeding follicular (18.2%; 2/11) and eccrine tumours (12.9%; 4/31); the odds ratio for sebaceous versus eccrine concordance was 28.0 (95% CI: 2.34-335). The anatomical site was the second significant predictor (χ² = 7.881, df = 3, p = 0.049), with head and neck tumours yielding the highest concordance (38.1%; 8/21) and trunk lesions an intermediate rate (20.0%; 2/10); tumours at both the upper and lower extremities were uniformly discordant (0%; 0/8 each). Sex, biological behaviour, pain, and the number of lesions were not significantly associated with concordance (all p > 0.05) (Table 3).

**Table 3:** Association of clinicopathological variables with diagnostic concordance.

Variable	Concordant (n=10) n (%)	Discordant (n=37) n (%)	Statistical Test	Test Statistic/OR (95% CI)	p-value
Eccrine	4 (12.9%)	27 (87.1%)	Chi-square (df=2)	χ² = 11.655 OR Sebaceous vs. Eccrine = 28.0 (95% CI: 2.34–335)	0.003
Follicular	2 (18.2%)	9 (81.8%)			
Sebaceous	4 (80.0%)	1 (20.0%)			
Benign	9 (23.1%)	30 (76.9%)	Fisher's Exact	OR = 1.26 (95% CI: 0.12–13.00)	1
Malignant	1 (12.5%)	7 (87.5%)			
Anatomical Site: Head & Neck	8 (38.1%)	13 (61.9%)	Chi-square (df=3)	χ² = 7.881	0.049
Anatomical Site: Trunk	2 (20.0%)	8 (80.0%)			
Anatomical Site: Upper Extremity	0 (0.0%)	8 (100.0%)			
Anatomical Site: Lower Extremity	0 (0.0%)	8 (100.0%)			
Pain: Present	2 (18.2%)	9 (81.8%)	Fisher's Exact		1
Pain: Absent	8 (22.2%)	28 (77.8%)			
Number of Lesions: Single	8 (22.9%)	27 (77.1%)	Fisher's Exact		0.706
Number of Lesions: Multiple	2 (16.7%)	10 (83.3%)			

Pattern of concordant diagnoses

Of the 10 concordant cases (21.3%; 95% CI: 9.6-33.0%), five were exact matches, and five were clinically reasonable approximations. Exact matches included trichoepithelioma, syringoma, and nevus sebaceous. Reasonable approximations included sebaceous adenoma labelled as sebaceous cyst, trichilemmal carcinoma submitted as squamous cell carcinoma, and malignant eccrine poroma labelled as eccrine poroma. Concordant cases were disproportionately from the sebaceous lineage and from entities with distinctive clinical phenotypes. The 37 discordant cases (78.7%) were predominantly eccrine tumours misidentified as sebaceous cysts, lipomas, implantation dermoids, or soft tissue sarcomas, or cases for which no clinical impression was provided (Table 4).

**Table 4:** Concordant case listing (n = 10; 21.3%; 95% CI: 9.6 – 33.0%)

S. No.	Histopathological Diagnosis	Clinical Diagnosis	Differentiation	Notes
1	Trichoepithelioma	Trichoepithelioma	Follicular	Exact match
2	Sebaceous adenoma	Sebaceous cyst	Sebaceous	Clinically reasonable approximation
3	Naevus sebaceous with syringocystadenoma	Naevus	Eccrine	Clinically reasonable approximation
4	Trichilemmal carcinoma	Squamous cell carcinoma	Follicular	Clinically reasonable approximation
5	Syringoma	Syringoma	Eccrine	Exact match
6	Syringoma	Syringoma	Eccrine	Exact match
7	Malignant eccrine poroma	Eccrine poroma	Eccrine	Clinically reasonable approximation
8	Nevus sebaceous	Nevus sebaceous	Sebaceous	Exact match
9	Nevus sebaceous	Nevus sebaceous	Sebaceous	Exact match
10	Nevus sebaceous	Nevus sebaceous	Sebaceous	Exact match

## Discussion

This study provides an integrated clinicopathological characterisation of 47 skin adnexal tumours over six years, encompassing histopathological spectrum analysis, clinicopathological associations, and systematic concordance quantification. The findings corroborate several established observations while contributing novel data.

Demographic profile

The male predominance (M:F = 1.47:1) is consistent with multiple Indian institutional series [4,5,7]. The classical female predominance of syringoma was confirmed, as all three syringoma cases in this cohort were female [15,16]. The peak incidence in the 41-60-year age group (44.7%) aligns with the fourth-fifth decade predominance reported by Sharma et al. [9] and Pujani et al. [7]. The wide age range (2-75 years) reflects the biological diversity of this tumour group, with the youngest case being a pilomatricoma in a two-year-old, consistent with its well-established paediatric predilection [15], and the oldest an eccrine tumour at 75 years.

Histopathological spectrum

Eccrine predominance (66.0%) is consistent with published Indian series, in which eccrine tumours have been reported in 61.2-64.5% of cases [5,6], attributable to the greater numerical density of eccrine glands across all body surfaces [1-3]. The hidradenoma group is the most frequent individual diagnosis (n=8; 17.0%; 95% CI: 8.9-30.1%), followed by pilomatricoma (n=5; 10.6%). The malignancy rate of 17.0% falls within the 10-25% range reported for tertiary referral series [4,9], reflecting referral bias toward atypical and recurrent lesions. All malignancies were of eccrine (6/8) or follicular (2/8) origin, mirroring the established pattern in Indian literature [4-7]. The absence of malignant sebaceous tumours is consistent with the documented rarity of sebaceous carcinoma and Muir-Torre syndrome-associated neoplasms in the Indian subcontinent relative to Western cohorts [17].

Anatomical site and differentiation distribution

Head and neck predominance (44.7%) is consistently reported in adnexal tumour series and is attributable to the high density of pilosebaceous and eccrine structures in the craniofacial region, compounded by greater cosmetic awareness, which prompts earlier presentation [2-7]. Eccrine tumours constituted the dominant lineage at every anatomical site (50-71%), though follicular tumours formed a relatively larger proportion at the extremities (25-38%) compared to the head and neck or trunk (19-20%). Despite these distributional differences, the association between the anatomical site and differentiation type was not statistically significant (χ² = 6.311, df = 6, p = 0.390), and anatomical location was similarly not associated with biological behaviour (p = 0.950), reinforcing that site alone cannot predict malignancy and that histopathological confirmation is mandatory irrespective of location [6,7].

Clinicopathological associations with biological behaviour

The absence of significant associations between any clinicopathological variable and biological behaviour is an important negative finding, consistent with Pujani et al. [7] and Kaur et al. [6], who similarly identified no reliable clinical predictors of malignancy in adnexal tumours. The non-significant trend toward higher pain prevalence in malignant tumours (50.0% vs. 17.9%) is biologically plausible, reflecting potential perineural infiltration, but the limited malignant sample (n=8) precludes definitive conclusions. Collectively, these findings reinforce the primacy of histopathological evaluation for all excised adnexal lesions, regardless of clinical presentation [1,3].

Diagnostic concordance

The overall concordance rate of 21.3% (95% CI: 9.6-33.0%) with Cohen's κ = −0.575 (95% CI: −0.792 to −0.357) represents the primary novel contribution of this study and, to our knowledge, the first systematic Kappa-based concordance analysis of the full adnexal tumour spectrum from an Indian tertiary centre. The negative kappa indicates agreement significantly worse than chance per the Landis and Koch classification [11], a finding rendered statistically robust by a confidence interval lying entirely below zero. This degree of clinical diagnostic failure exceeds the 'fair' to 'moderate' concordance documented for other skin tumours such as basal cell carcinoma and melanocytic lesions, even without dermoscopy [13,18], highlighting the exceptional diagnostic opacity of adnexal tumours. The most common driver of discordance was misidentification as a sebaceous cyst, applied to eccrine spiradenoma, chondroid syringoma, pilomatricoma, and multiple cases within the Hidradenoma group, reflecting the clinical non-specificity of smooth, mobile, dermal nodules without surface changes [4-7,15]. Notably, the Hidradenoma group achieved 0% concordance despite being the most frequent entity, underscoring that high institutional frequency does not translate into greater clinical recognition. Malignant tumours were not more likely to be correctly identified than benign ones (25.0% vs. 23.1%; p = 1.000), confirming that histological malignancy confers no clinical diagnostic advantage for lineage-specific identification [2,3,15].

Predictors of concordance

Sebaceous differentiation was the strongest predictor of concordance (80.0%; OR = 28.0 vs. eccrine; 95% CI: 2.34-335; p = 0.003), explained by the clinically distinctive phenotypes of sebaceous entities: Nevus Sebaceous of Jadassohn presents as a characteristic yellowish-orange verrucous plaque in young patients [16], and sebaceous hyperplasia as an umbilicated yellowish papule in middle-aged individuals [17,18], both with well-defined site predilections that facilitate clinical recognition. Head and neck location was the second significant predictor (38.1% vs. 0% for extremities; p = 0.049), likely reflecting cosmetic visibility, clinician familiarity with craniofacial adnexal entities, and the concentration of clinically distinctive sebaceous and follicular tumours at this site. The uniformly zero concordance at both extremity sites, where hidradenomas, poromas, and spiradenomas present as non-specific nodules indistinguishable from non-adnexal lesions [2,3], carries a direct practical implication: excised skin nodules from extremity locations must be regarded as diagnostically opaque to clinical assessment, and thorough histopathological evaluation is non-negotiable [1-3].

Strengths and limitations

Strengths include consecutive total case enumeration, structured pro forma-based data collection, WHO-based histopathological classification with multidisciplinary review, and Kappa-based concordance quantification. The primary limitation is the sample size (n=47), which limits power for subgroup analyses of malignancy and precludes multivariate concordance modelling.

## Conclusions

This six-year institutional analysis confirms eccrine predominance (66.0%) and a benign majority (83.0%) in skin adnexal tumours, with Hidradenoma emerging as the most frequent consolidated diagnosis. The striking 78.7% clinical-histopathological discordance rate (Cohen’s κ = −0.575) unequivocally establishes histopathological examination as indispensable for all excised adnexal lesions, irrespective of clinical impression. Sebaceous differentiation and head-and-neck location were independent concordance predictors, whereas extremity lesions were uniformly discordant, mandating heightened diagnostic vigilance at these sites. These findings provide clinically actionable, region-specific evidence supporting routine and systematic histopathological evaluation of every excised skin adnexal lesion.
